# SARS-CoV-2 helicase might interfere with cellular nonsense-mediated RNA decay: insights from a bioinformatics study

**DOI:** 10.1186/s12863-023-01173-y

**Published:** 2023-11-18

**Authors:** Behnia Akbari, Ehsan Ahmadi, Mohammad Reza Zabihi, Mina Roshan Zamir, Mina Sadeghi Shaker, Farshid Noorbakhsh

**Affiliations:** 1 Department of Immunology, School of Medicine, Tehran University of Medical Sciences, Tehran, Iran; 2 Shefa Neuroscience Research Center, Khatam Alanbia Hospital, Tehran, Iran

**Keywords:** SARS-CoV-2, Coronaviridae, Helicase, RNA surveillance, Nonsense-mediated RNA decay

## Abstract

**Background:**

Viruses employ diverse strategies to interfere with host defense mechanisms, including the production of proteins that mimic or resemble host proteins. This study aimed to analyze the similarities between SARS-CoV-2 and human proteins, investigate their impact on virus-host interactions, and elucidate underlying mechanisms.

**Results:**

Comparing the proteins of SARS-CoV-2 with human and mammalian proteins revealed sequence and structural similarities between viral helicase with human UPF1. The latter is a protein that is involved in nonsense-mediated RNA decay (NMD), an mRNA surveillance pathway which also acts as a cellular defense mechanism against viruses. Protein sequence similarities were also observed between viral nsp3 and human Poly ADP-ribose polymerase (PARP) family of proteins. Gene set enrichment analysis on transcriptomic data derived from SARS-CoV-2 positive samples illustrated the enrichment of genes belonging to the NMD pathway compared with control samples. Moreover, comparing transcriptomic data from SARS-CoV-2-infected samples with transcriptomic data derived from UPF1 knockdown cells demonstrated a significant overlap between datasets.

**Conclusions:**

These findings suggest that helicase/UPF1 sequence and structural similarity might have the ability to interfere with the NMD pathway with pathogenic and immunological implications.

**Supplementary Information:**

The online version contains supplementary material available at 10.1186/s12863-023-01173-y.

## Background

The proteome of SARS-CoV-2, the virus responsible for the COVID-19 pandemic, consists of a wide array of proteins, including both structural and non-structural proteins (nsp). These proteins play crucial roles in the virus’ life cycle, encompassing key processes such as cell entry, viral replication, and gene expression [1, 2]. In addition to harboring elements necessary for their life cycle, viruses are known to possess remarkable capabilities to interfere with host defense mechanisms. These mechanisms are quite diverse, with some viruses interfering in immunological defense mounted by leukocytes, while others inhibiting innate factors that restrict viral replication within target cells. One strategy employed by viruses to influence anti-viral defense mechanisms is the production of proteins that exhibit sequence or structural similarities with host proteins. In the case of DNA viruses, which have relatively large genomes, this phenomenon is known as “gene hijacking” [3]. Gene hijacking occurs during viral evolution when viral genomes acquire host genes through processes like retrotransposition [4]. This results in the production of proteins that resemble their host counterparts, allowing viruses such as herpesviruses and other large DNA viruses to manipulate host defense mechanisms [3, 4]. However, for viruses with smaller genomes, and hence lower capacity to incorporate entirely new genes, alternative mechanisms are employed to enable viral proteins to mimic host proteins [5]. These mechanisms involve exploiting similarities in protein sequence or structure, allowing the viral proteins to mimic and potentially interfere with their host counterparts [5]. In this study we sought to identify potential similarities between SARS-CoV-2 proteins with human proteins and investigate whether these similarities might possibly influence virus-host interactions. To accomplish this, we conducted a comprehensive comparison of all SARS-CoV-2 protein sequences with reference protein sequences from humans. Subsequently, we analyzed the structural similarities between a subset of these proteins. Furthermore, we leveraged transcriptomic data derived from cells and tissues infected with SARS-CoV-2 to investigate whether the observed similarities might impact host anti-viral mechanisms. Exploring the ways by which SARS-CoV-2 proteins mimic human proteins, our study provides insights into the intricate interactions between the virus and its host. Understanding these interactions is crucial for the development of effective therapeutic strategies and the identification of potential drug targets. Moreover, these findings contribute to the broader understanding of viral pathogenesis and shed light on the sophisticated strategies employed by viruses to evade and manipulate host defenses.

## Results

### SARS-CoV-2 proteins show sequence similarities with human and mammalian proteins

**Fig. 1:**
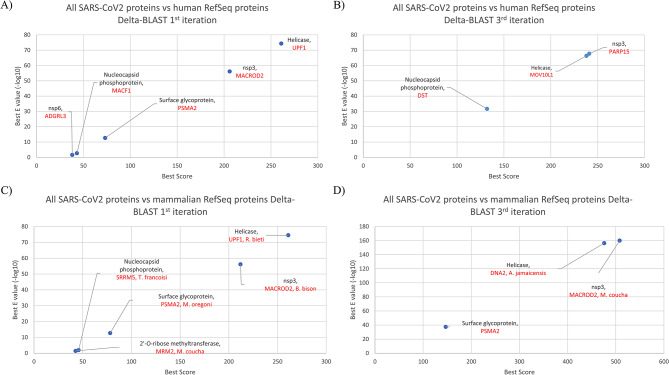
SARS-CoV-2 Nsp3 and helicase reveal higher sequence similarities with human/mammalian proteins. Results of the 1st (**A**) and 3rd (**B**) iterations of DELTA-BLAST comparing SARS-CoV-2 protein sequences with *Homo sapiens* RefSeq proteins have been shown as negative log E values/alignment scores. Likewise, results of the 1st (**C**) and 3rd (**D**) iterations of DELTA-BLAST comparing SARS-CoV-2 proteins against mammalian RefSeq proteins are shown. Below every SARS-COV-2 protein, the name of the aligned human or mammalian protein (together with the species for the latter) is shown with red fonts. For each query only the highest log E value/score has been shown

In order to find potential protein sequence similarities between SARS-CoV-2 and *Homo sapiens*/ mammalian proteins, SARS-CoV-2 reference protein sequences (RefSeq) were extracted from NCBI protein database (Table S1). DELTA-BLAST (Domain Enhanced Lookup Time Accelerated Basic Local Alignment Search Tool) was used to compare all SARS-CoV-2 RefSeq protein sequences against reference sequences of *Homo sapiens* and mammalian proteins. As shown in Fig. 1, SARS-CoV-2 non-structural protein 3 (nsp3) showed significant similarities (based on e value) with both human and mammalian proteins (matched with MACROD2 in iteration 1 and PARP15 in iteration 3). Interestingly, several groups have previously reported that nsp3 of coronaviruses interferes with interferon signaling pathway, possibly via the reversal of protein ADP-ribosylation, a posttranslational modification catalyzed by host poly (ADP-ribose) polymerases (PARPs) [6]. Another significant result was detected for SARS-CoV-2 helicase protein (non-structural protein 13) when aligned with either human or mammalian Refseq proteins. Viral helicase showed the highest similarities with UPF1 and Mov10L1 in humans and DNA2 in mammals, proteins which have helicase activity. Less significant similarities were observed between viral nucleocapsid phosphoprotein (with human MACF1 and Dystonin isoform 1eA precursor and mammalian SRRM5), viral surface glycoprotein (with human PSMA2), and viral nsp6 (with ADGRL3 in humans and MRM2 in mammals) and human/mammalian proteins (Fig. 1A-D).

**Fig. 2:**
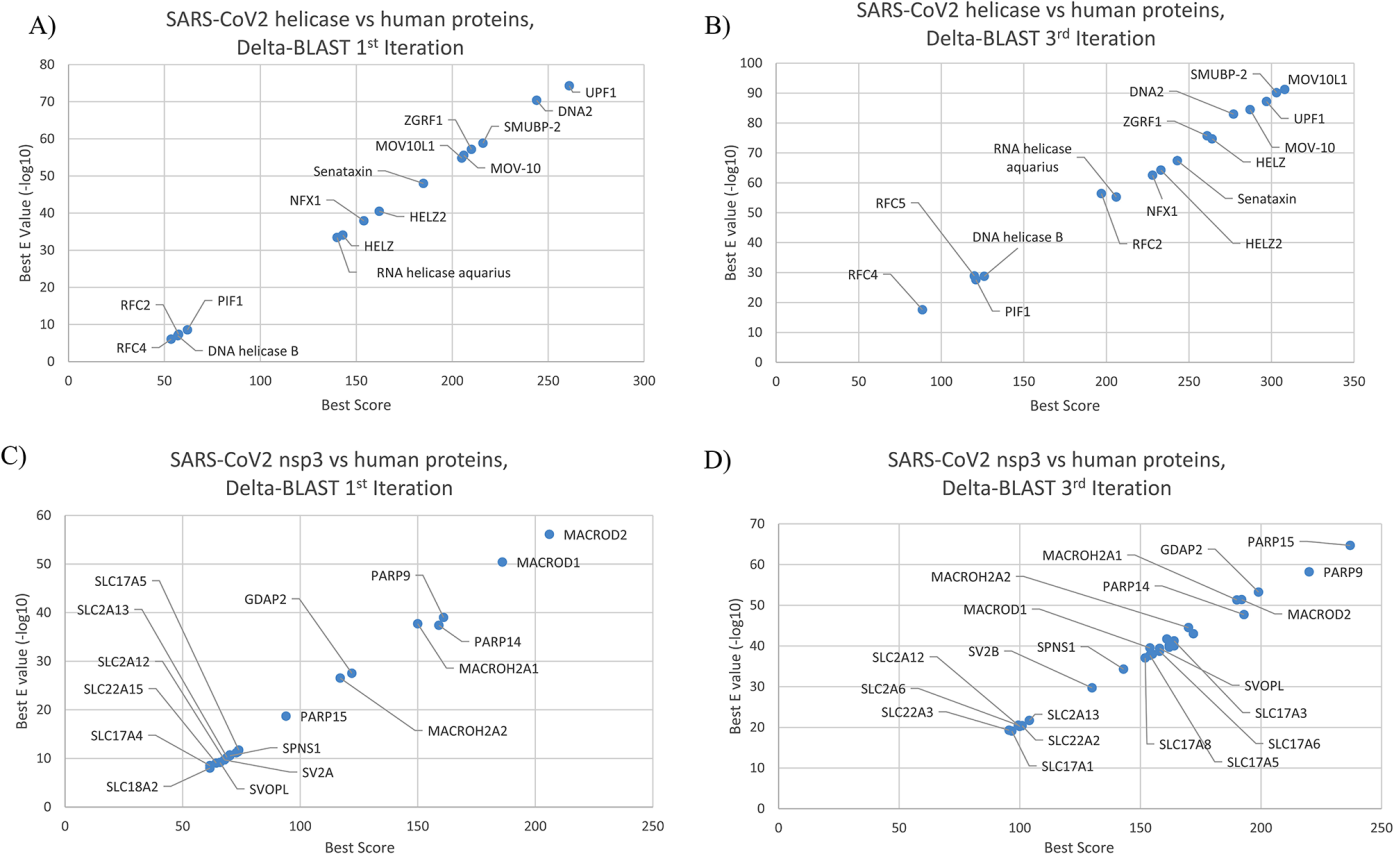
SARS-CoV-2 helicase protein sequence similarities with human proteins. Results of the 1st and 3rd iterations of DELTA-BLAST comparing SARS-CoV-2 helicase (**A** and **B**) or nsp3 (**C** and **D**) with *Homo sapiens* RefSeq proteins

Focusing on nsp3 and helicase, we examined all human proteins that showed sequence similarities with these two proteins. Viral helicase (YP_009725308.1) exhibited higher degrees of sequence similarity with human UPF1 (UPF1 RNA helicase and ATPase), DNA2 (DNA replication ATP-dependent helicase/nuclease), MOV10L1 (Mov10 like RISC complex RNA helicase 1) and SMUBP2 (DNA-binding protein SMUBP-2), which are all members of helicase superfamily (Fig. 2A and B) [7]. Of these proteins, UPF1 has known roles in nonsense-mediated RNA decay (NMD), a surveillance mechanism for mRNAs containing premature termination codons, as well as viral RNAs (Figure S1) [8]. For viral nsp3, highest degree of similarities were observed with human MACROD1, MACROD2 as well as PARP9/15 (poly ADP-ribose polymerase family members). These proteins are mainly known as members of macro domain protein family that can bind to ADP-ribose [9] (Fig. 2C and D).

### Helicase domain and CH domains of viral nsp13 show structural similarities with UPF1

**Fig. 3:**
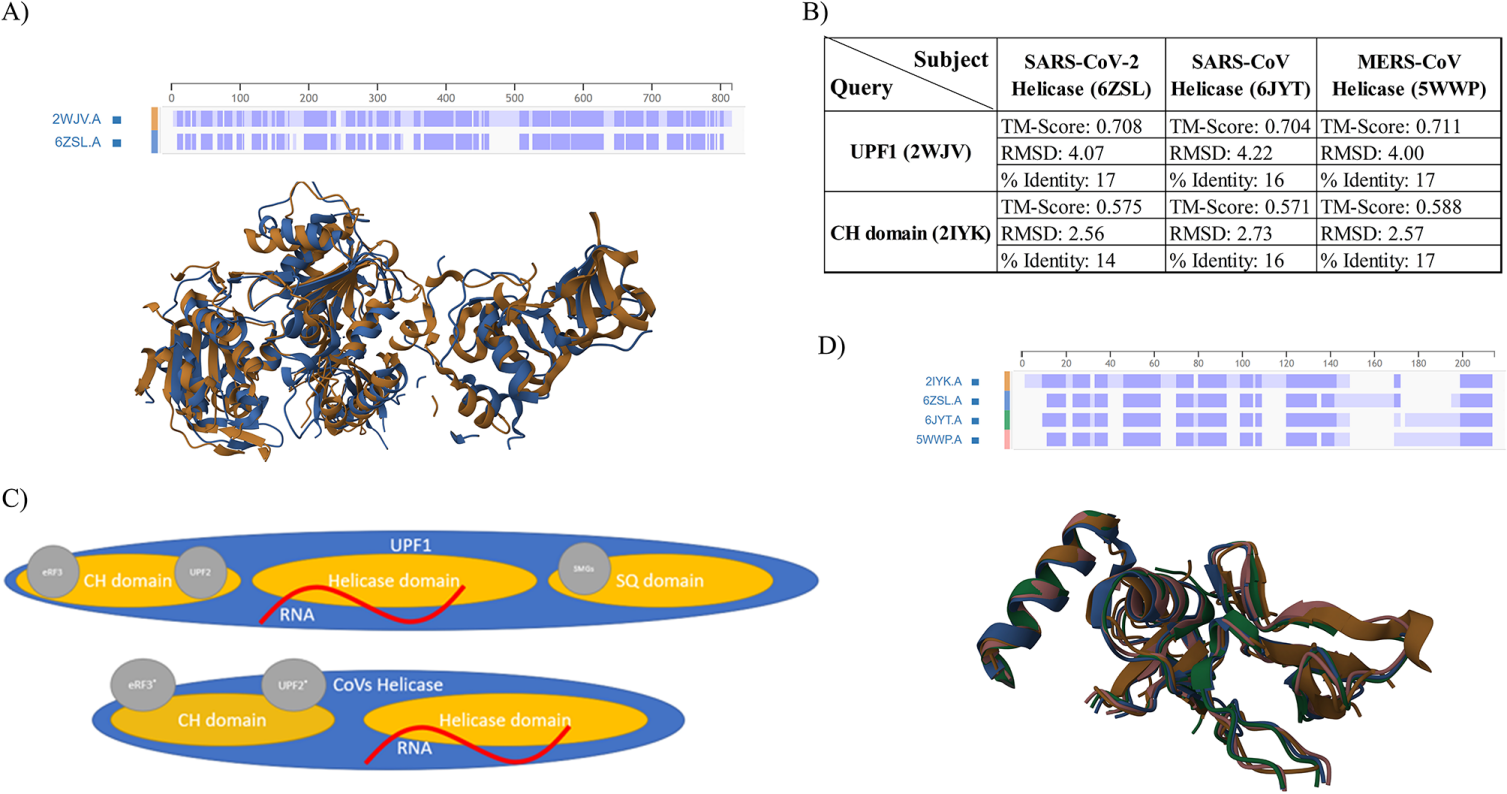
Structural comparison and schematic representation of domains of human UPF1 and coronaviral helicases. Representation of pairwise structure alignment of SARS-CoV-2 with UPF1 (**A**). Data extracted from pairwise structure alignment of SARS-CoV-2, SARS-CoV, and MERS-CoV helicase protein with UPF1 and CH domain (**B**). Schematic representation of domains of human UPF1 and coronaviral helicases (**C**). Pairwise structure alignment of SARS-CoV-2, SARS-CoV, and MERS-CoV with UPF1 CH domain (**D**). RMSD: Root Mean Square Deviation

To determine if the observed sequence similarities between SARS-CoV-2 helicase and human proteins also corresponded to structural similarities, the structure of SARS-CoV-2 helicase (PDB/6ZSL) was compared with the structure of human UPF1 (PDB/2WJV) using RCSB “Pairwise Structure Alignment” and “TM-align” [10, 11]. This structural comparison yielded a TM-Score of 0.708, and RMSD value of 3.15 (Fig. 3A). The helicase structures of other highly pathogenic coronaviruses, SARS-CoV and MERS-CoV, were also compared with human UPF1 [12, 13], showing high TM-score/RMSD values, indicating that this similarity is not limited to SARS-CoV-2 helicase (Fig. 3B). More specifically, The UPF1 protein consists of three domains: CH, Helicase, and SQ domains (Fig. 3B). The CH domain interacts with UPF2, while the SQ domain inhibits helicase activity. Viral helicases lack the inhibitory SQ domain, containing only helicase and CH domains (Fig. 3C) [14]. The CH domain of UPF1 inhibits its catalytic activity, which is relieved upon binding of the CH domain to UPF2, another member of the NMD pathway. Hence, UPF1/UPF2 interaction via UPF1’s CH domain is a critical step in initiation of NMD pathway. To find out whether UPF1 CH domain has any structural similarities to its corresponding domain in viral helicase, we conducted pairwise structure alignment analysis of the crystal structure of the CH domain of UPF1 (PDB/2IYK) with viral helicase structures [15]. As illustrated in Fig. 3B and D, the sequence similarities and TM-Score between all three viral helicases and the UPF1 CH domain were somewhat consistent with the earlier comparisons involving UPF1 and viral helicases. Furthermore, to better investigate the potential of viral helicase to interact with UPF2, we identified the amino acids involved in UPF1-UPF2 interaction from the UPF1-UPF2 complex crystal structure (PDB/2WJV) using LigPlot (REF). We then structurally aligned UPF1 with viral helicase (PDB/6ZSL) using TM-align and located those amino acids in the alignment. Among the nine amino acids involved in UPF1-UPF2 interaction, only one amino acid was identical in both proteins. However, a total of six out of the nine amino acids were also aligned between UPF1 and viral helicase in the structural analysis (Figure S2).

### SARS-CoV-2 infection leads to altered expression of NMD pathway transcripts

NMD is a highly conserved pathway which exists in all eukaryotic organisms. NMD’s key function is to remove mRNAs transcribed from genes with nonsense mutations, hence preventing these mRNAs from producing incomplete/truncated proteins [16]. This is performed by detection of premature termination codons (PTCs) in mRNA transcripts [17]. That said, NMD has also been shown to target other ‘unwanted’ RNAs, including viral RNAs [18]. While viral RNAs do not contain PTCs per se, the multi-cistronic nature of viral genomes makes their transcripts susceptible to NMD, as the internal stop codons are recognized as PTCs by the NMD pathway [8]. Interestingly, different viruses have developed mechanisms to circumvent the targeting of their RNAs by NMD [19, 20]. Considering the protein sequence/structure similarities that we observed between SARS-CoV-2 helicase and human UPF1, we asked whether this might lead to any interference with host cells NMD pathway. To check whether NMD pathway members are affected in SARS-CoV-2-infected cells/tissues, we decided to analyze RNA sequencing data derived from cells/tissues infected with the virus. We extracted raw data from datasets deposited in NCBI GEO, i.e., GSE155974, GSE171110, GSE157103 and GSE182917. Of these, GSE155974 contains transcriptomic data from in vitro infected cells, GSE171110 and GSE157103 contains transcriptomic data of COVID-19 and healthy patients, and GSE182917 contains data from lung autopsy tissue (details are shown in Table S3). To investigate whether viral infection has influenced NMD pathway, we performed Gene Set Enrichment Analysis (GSEA) on these datasets, as described in the Methods section.

**Fig. 4:**
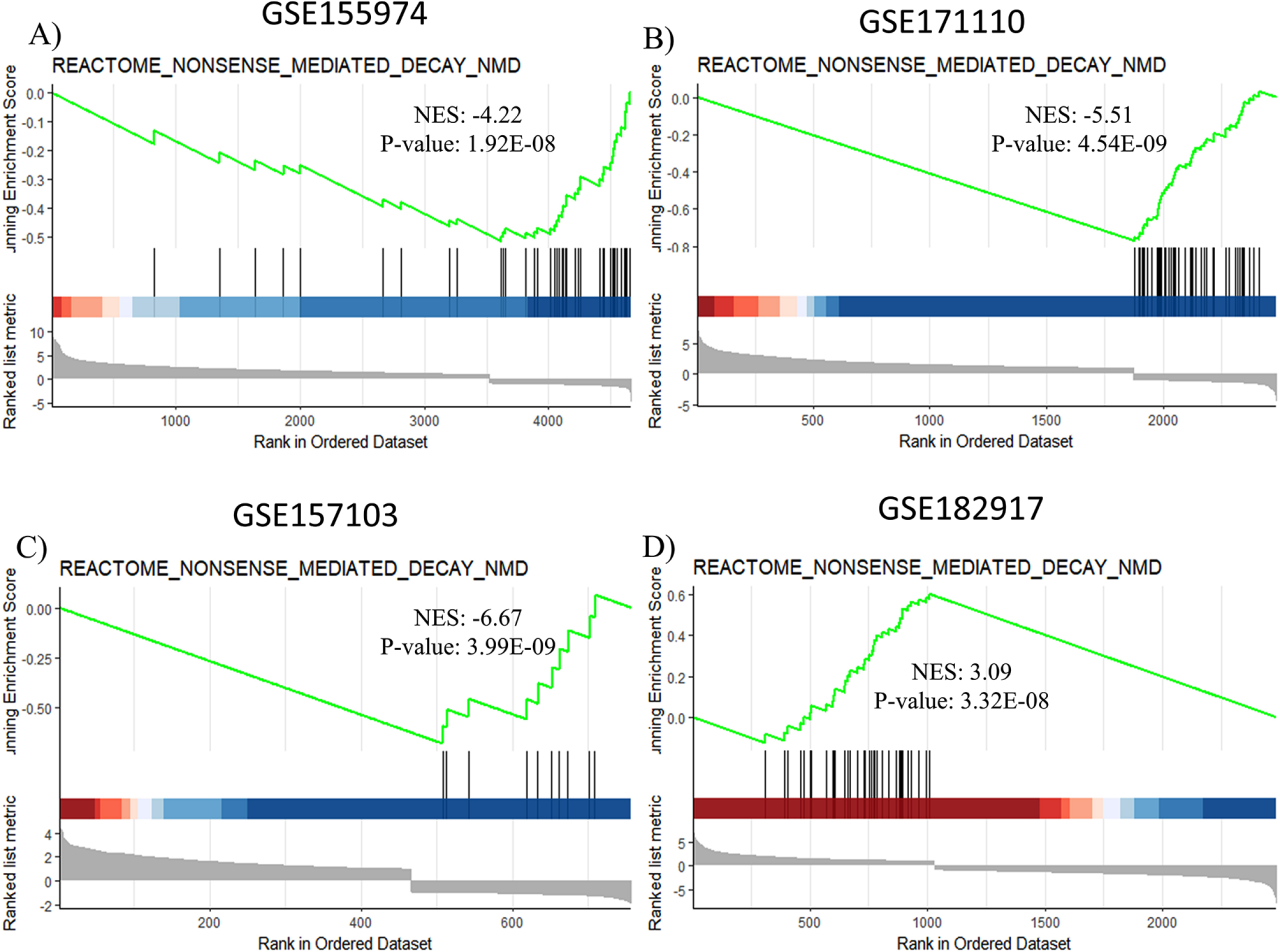
GSEA analyses on different SARS-CoV-2-infected cells/tissues. GSEA analyses of GSE155974 (**A**), GSE171110 (**B**), and GSE157103 (**C**) GSE182917 (**D**). All GSEA analyses, except one, show negative enrichment of NMD pathway in samples infected with SARS-CoV-2

Our analyses showed negative enrichment of NMD pathway gene set members in the first three datasets; i.e., infected nasal organoid cells (Fig. 4A), whole blood (Fig. 4B) and leukocytes (Fig. 4C) derived from COVID19 patients. In contrast, the last dataset; i.e., lung autopsy tissues, showed a positive enrichment for NMD gene set (Fig. 4D). This apparent contradiction can be attributed to the distinct viral infection and replication mechanisms that operate across different cell types. NMD pathway members are known to restrict viral replication [21], and downregulation of NMD pathway elements has been associated with higher levels of viral proteins in infected cells [17]. We believe these data suggest potential suppression of NMD pathway activity in SARS-CoV-2-infected cells. Nonetheless, these transcriptional changes do not necessarily reflect perturbed UPF1 function caused by viral proteins; e.g., nsp13.

### SARS-CoV-2-induced transcriptional changes show a significant overlap with transcriptional changes induced by UPF1 knockdown

Perturbed activity of NMD pathway leads to increased expression of its target transcripts. While, PTC-containing transcripts represent an important group of NMD targets, studies have shown that numerous normal transcripts are also recognized and targeted by NMD [17]. Presence of features like uORFs, long 3’UTRs and some other non-PTC elements can make the transcripts susceptible to NMD degradation. In an effort to systematically identify NMD target transcripts, Colombo et al. performed transcript profiling on cells in which different NMD players were knocked down [22]. Their results showed that the majority of NMD’s ‘normal’ targets were protein-coding genes, followed by pseudogenes and non-coding RNAs. Considering the availability of these data, we asked whether transcriptional changes caused by SARS-CoV-2 infection, had any overlap with transcriptional alterations observed by Colombo et al. following the knockdown of NMD elements.

**Fig. 5:**
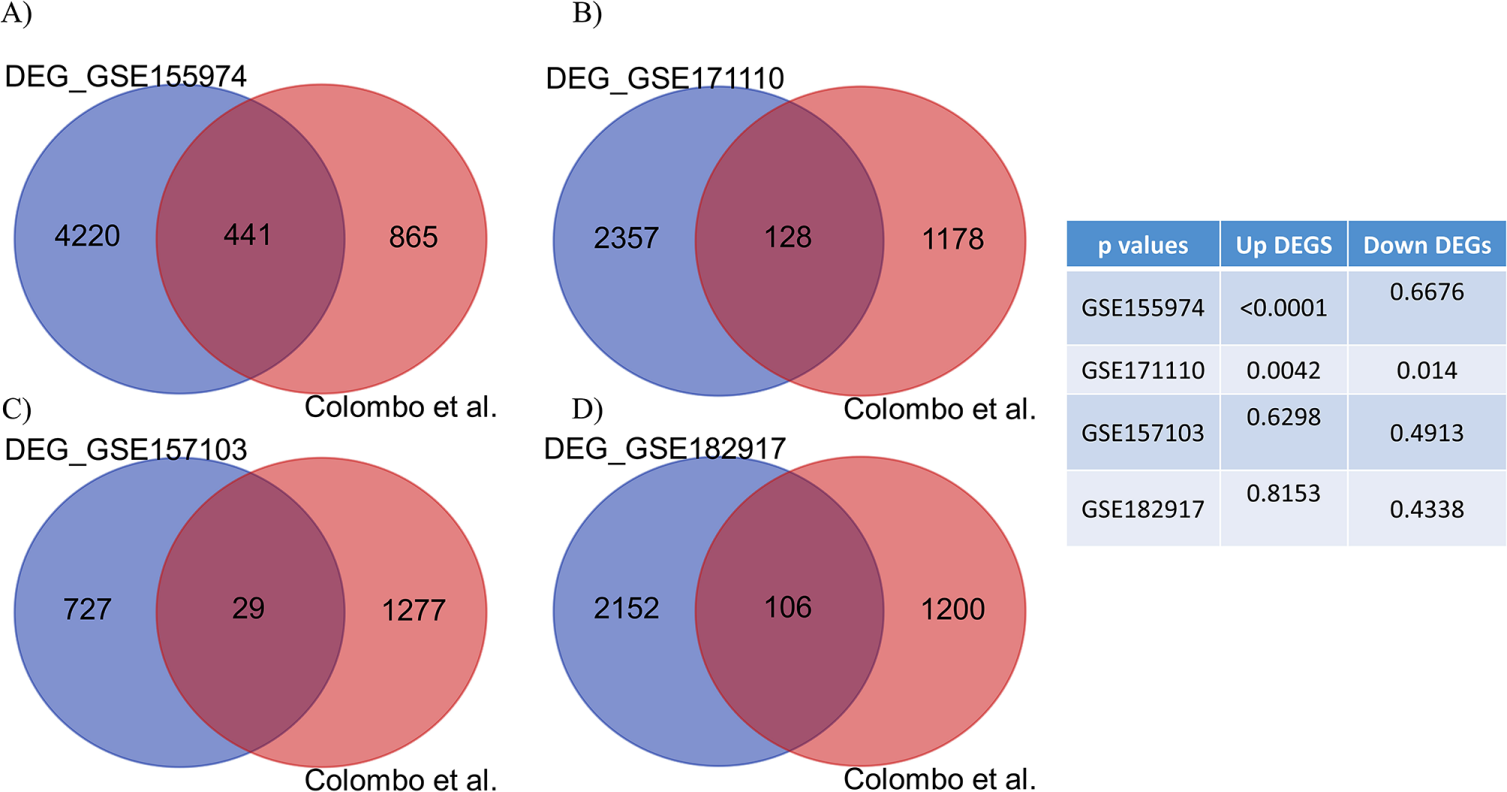
Venn diagrams showing the similarity in DEGs between Colombo et al. study and SARS-CoV-2-infected cells/tissues. Transcripts showing significant alterations (FDR < 0.05) between Colombo et al. study and GSE155974 (**A**), GSE171110 (**B**), and GSE157103 (**C**) GSE182917 (**D**) studies are shown. Table shows p values for Up and down-DEGs shared by the two studies

To this end, we compared the list of upregulated and down-regulated DEGs in Colombo et al. dataset with SARS-CoV-2-infected cells/tissues (Table S4). As shown in Fig. 5, a significant overlap, particularly in the upregulated genes, was observed between Colombo et al. dataset and GSE155974 as well as GSE171110. Additionally, Gene Ontology analysis indicated the involvement of these overlapping genes (Table S4) in various processes, such as glycerophospholipid and phosphatidylinositol biosynthetic processes, cholesterol import and homeostasis, and positive regulation of the apoptotic process (Figure S3). Notably, SMG1, a component and regulator of the NMD pathway, is a phosphatidylinositol 3-kinase-related protein kinase involved in cholesterol homeostasis [23, 24]. Furthermore, previous research has shown that attenuation of NMD pathway proteins can induce programmed cell death and cellular apoptosis [25, 26]. While these are indirect evidences, they likely reflect alterations induced by SARS-CoV-2 in NMD pathway activity.

## Discussion

Nonsense-mediated RNA decay (NMD) is known as an mRNA surveillance pathway that controls gene expression quality by recognizing and removing ‘faulty’ transcripts. NMD was initially discovered as an mRNA degradation pathway that detected transcripts with premature termination codons (PTCs) [27–29]. Further studies revealed that NMD targets might have features other than PTCs; i.e., presence of long 3’ untranslated regions (UTR), upstream open reading frame (uORF) or termination codons which ‘mimic’ PTCs. NMD initiation requires a set of evolutionarily conserved proteins including up-frameshift protein 1 to 3 (UPF1-3) as well as SMG1/SMG5-9 which are involved in RNA cleavage and degradation [30, 31] and polypeptide chain release factors also known as eRFs [16, 32–34].

UPF1 is a helicase that is known as the master regulator of NMD in cells [35]. It is also involved in other processes including DNA repair and replication, telomere length homeostasis, mRNA transport, and RNA localization. This protein consists of three main regions; an N-terminal cysteine-histidine rich (CH) domain which binds to UPF2, ribosomal protein S26 (RPS26), and mRNA-decapping enzyme 2 (DCP2), a helicase domain that binds to single-stranded RNA (ssRNA) and DNA (ssDNA) molecules and a nonstructural serine-glutamine (SQ)-rich C-terminal domain which interact with SMGs (suppressor with morphogenetic defects in genitalia) [14, 36–39].

During translation of ‘normal’ transcripts, eukaryotic release factor 1 (eRF1) is required for recognition of stop codons and termination of translation. Following the recognition of stop codon by eRF1, another protein named eRF3 binds to eRF1 and facilitates the release of the nascent polypeptide from the ribosome through its GTPase activity [40]. Another player is cytoplasmic polyA-binding protein 1 (PABPC1) which binds to mRNA polyA tails and enhances the recruitment of eRFs [41]. During translation, movement of ribosomes across the transcripts leads to the displacement and removal of exon junction complex (EJC) proteins which are normally present at EJCs. In an aberrant mRNA translation termination; e.g., when the ribosome reaches a termination codon which is located upstream of an EJC, the presence of unremoved EJC proteins leads to the recruitment of UPF proteins to the location. UPF1 is recruited via UPF2 to EJC-UPF3B complex and is phosphorylated by SMGs, which finally results in NMD pathway execution [34, 42]. NMD activation might also happen in the absence of EJC proteins. In one of the mechanisms reported for EJC-independent NMD activation, the transcript’s long 3’-UTR can prevent PABPC1 from interacting with eRF3 and instead allowing UPF1 interaction to eRF3 [43]. Hence, the competition between PAPBC1 and UPF1 for binding to eRF3 can change the mRNA survival [42, 44].

Many viruses use long RNA transcripts containing multiple ORFs during their gene expression and life cycle. These long RNAs generally contain several termination codons inside the transcript and far from the polyA tail. This feature mimics the presence of PTCs in eukaryotic transcripts and can lead to the activation of NMD pathway and degradation of viral transcripts. That said, it has been shown that viruses employ different mechanisms to protect their transcripts from the NMD pathway [34]. Semliki Forest virus (SFV) and Sindbis virus (SINV) are alphaviruses from togaviridae family which carry positive-sense ssRNA with long 3’-UTRs. Genome-wide small interfering RNA (siRNA) studies used to identify host factors involved in viral replication have shown that UPF1 and other NMD factors act as restrictors for the replication of these viruses by degrading their RNAs [45, 46]. A combination of proteomics and RNAi screening approaches on hepatitis C virus (HCV) infected cells have also revealed that the core protein of HCV inhibits NMD and increases virus replication in hepatoma cell lines [47]. NMD-inhibiting mechanisms have also been reported for retroviruses. Rous Sarcoma Virus (RSV) has a 400-nucleotide long sequence downstream of its gag mRNA called RNA stability element (RSE) which can form an RNA secondary structure and prevent the recruitment of UPF1 and the execution of NMD [48, 49]. Moreover, tax and rex, two proteins from Human T-lymphotropic Virus Type 1 (HTLV-1) have been shown to bind UPF1 and prolong viral genome half-life by preventing their RNA from degradation [50, 51]. A few studies have demonstrated interactions between HIV-1 with UPF proteins [52–54]. There is evidence that anti-NMD mechanisms might also exist for coronaviruses. In a study by Wada et al researchers have shown that N protein of murine hepatitis virus (MHV), a member of the betacoronaviruses, may partially inhibit NMD execution [55]. Whether other MHV or coronaviral proteins can inhibit NMD is unclear.

In this study, we found significant sequence and structure similarity between SARS-CoV-2 helicase (nsp13 in other pathogenic β-CoVs) with human UPF1. We then used GSEA analysis of RNA-seq data derived from SARS-Cov-2-infected samples to see whether NMD-related genes show any alterations following viral infection. We then examined transcriptomic data from UPF1 knockdown cells to investigate whether any similarities might exist between these cells and virus infected samples at the molecular level. Our analyses provided some evidence, albeit indirect, for possible interference of viral helicase (nsp13) with the NMD pathway. It is conceivable that SARS-CoV-2 helicase could interfere with cellular NMD by interacting with UPF2 and eRF through its sequence and structural similarity with UPF1. Our findings are consistent with previous studies that have highlighted the resemblance between UPF1 and pathogenic β-CoVs and the potential of these viruses to escape the host’s immune system by mimicking UPF1 to interact with UPF2 [10, 12, 13]. However, it is essential to emphasize that further experimental evidence is required to establish a more conclusive understanding of these interactions.

## Conclusion

Taken together, our results suggest that SARS-CoV-2 helicase might interfere with cellular nonsense-mediated RNA decay pathway. However, further direct evidences are needed to approve our results.

## Methods

### Data extraction

Reference protein sequences of SARS-CoV-2 were extracted from NCBI protein database (https://www.ncbi.nlm.nih.gov/protein/). Polyprotein and redundant sequences were removed. All accession numbers and their official gene symbols were shown in Table S1. 3D structure of proteins (2IYK, 2XZO, 2XZP, 6JYT, 5WWP) were downloaded from Protein Data Bank (PDB: https://www.rcsb.org/). RNA sequencing datasets were obtained from NCBI Gene Expression Omnibus (GEO: https://www.ncbi.nlm.nih.gov/gds). Datasets in which cells or patients were under treatment were excluded. The raw count data were downloaded in .csv or .txt format.

### Sequence and structural alignments

Protein sequence similarity searches were performed for all SARS-CoV-2 proteins against human and mammalian reference protein sequences using NCBI DELTA-BLAST (Domain Enhanced Lookup Time Accelerated BLAST: blast.ncbi.nlm.nih.gov/Blast.cgi). All DELTA-BLASTs were followed by three iterations of PSI-BLAST (Position-Specific Iterative-BLAST). DELTA-BLAST parameters are shown in Table S2. To report the strongest similarities, highest E-value/alignment score pairs were extracted for each query. Results were visualized as negative Log10 of E-values and alignment score. Pairwise structural alignment for viral helicase proteins against UPF1 and CH domain of UPF1 was carried out using RCSB Pairwise Structure Alignment tool (www.rcsb.org/alignment) and TM-align (www.zhanggroup.org/TM-align) [56]. Additionally, we utilized LigPlot software to identify the amino acids involved in the interaction between UPF1 and UPF2 [57].

### PPI network

The search tool for retrieval of interacting genes (STRING) (https://string-db.org/, version 11.0) database were applied to predict functional interactions of proteins with UPF1. Active interaction sources, experiments, and databases, as well as species limited to “Homo sapiens” and an interaction score > 0.9 were applied to construct the PPI networks. Cytoscape software (version 3.6.1) was used to visualize the PPI network.

### Gene ontology analysis

Functional enrichment analysis was performed using the Gene Ontology (GO) database (http://geneontology.org), ClusterProfiler R package, and EnrichR [58–60]. GO term enrichment was done for biological processes (BP), molecular function (MF) and cellular components (CC) categories. Data were visualized using R ggplot2 package. Terms with adjusted p-value less than 0.05 reported significant.

### RNA-seq data processing

Datasets including RNA seq data from SARS-CoV-2-infected samples were extracted from NCBI GEO. Datasets were imported into R studio using built-in “read.csv” function. Digital gene expression lists were generated using edgeR package and “DEGList” function. Data filtration and normalization were performed using Trimmed Mean of M-values (TMM) method via “calcNormFactors” function in edgeR package. Differentially expressed genes (DEGs) were determined from each dataset. T-test were performed to assess differential expression of genes between COVID-19 samples and healthy controls. Benjamini Hochberg method were used for p-value adjustment. To determine the effect size for each gene, the mean ratio of each COVID-19 gene versus the average expression of it in healthy controls were calculated (Fold-Change). Genes with adjusted p-value < 0.05 and |log2 FC| > 1 were considered as DEGs. Furthermore, samples were annotated with NCBI official gene symbols using Homo sapiens annotation package (hgu133plus2.db). The biomaRt package were further used to match Ensembl gene IDs to official gene names extracted from hgu133plus2.db.

### Gene set enrichment analysis

DEGs were extracted from datasets according to the above-mentioned pipeline. In the next step, DEGs from each dataset were sorted according to their FC. GSEA analyses were performed using the “GSEA” function of clusterProfiler package in R software. C2 category of the Msigdb was used as gene sets in our GSEA. p-values less than 0.05 were considered to indicate significant enrichment. GSEA results were visualized with “gseaplot2” function of R software’s enrichplot package.

### Electronic supplementary material

Below is the link to the electronic supplementary material.

Supplementary Material 1. Table S1. SARS-CoV-2 proteins accession numbers and symbols

Supplementary Material 2. Table S2. Delta-BLAST parameters

Supplementary Material 3. Table S3. GEO Dataset attributes

Supplementary Material 4. Table S4. List of overlapping genes between DEGs_GSE155974, DEGs_GSE171110, DEGs_GSE157103, and DEGs_GSE182917 with genes extracted from Colombo et al study

Supplementary Material 5. Figure S1. Protein-protein interaction map for UPF1. UPF1 interacts with CASC3, UPF2, GSPT2, UPF3A, EIF4A3, GSPT1, UPF3B, SMG7, RBM8A, SMG1 in Homo sapiens (A). Gene Ontology Map showing important biological processes (BP), cellular components (CC), and molecular functions (MF) in cells (B). GO analysis of UPF1 and its interacting proteins revealed these proteins main biological process in cells is nonsense-mediated RNA decay

Supplementary Material 6. Figure S2. Amino acids involved in UPF1-UPF2 interaction. The LigPlot output reveals the presence of nine amino acids participating in the interaction between UPF1 and UPF2. UPF1 amino acids names are shown in blue (below the dotted line) and UPF2 amino acid names are shown in green (above the dotted line) (A). Structural alignment between UPF1 and viral helicase revealed that six out of the nine UPF1-UPF2 interface amino acids exhibited alignment with their counterpart aminoacids in the SARS-CoV-2 helicase (B)

Supplementary Material 7. Figure S3. Gene ontology analysis of overlapping genes with Colombo et al study. Bar graphs representing biological process of overlapping genes between DEGs_GSE155974 (A), DEGs_GSE171110 (B), DEGs_GSE157103 (C), and DEGs_GSE182917 (D) with genes extracted from Colombo et al study

## Data Availability

All data generated or analyzed during this study are included in this published article and its supplementary information files.

## List of abbreviations

NMD: Nonsense-mediated RNA decay
GO: Gene Ontology
BP: Biological process
MF: Molecular function
CC: Cellular component
SARS-CoV: Severe Acute Respiratory Syndrome coronavirus
MERS-CoV: Middle East respiratory syndrome–related coronavirus
